# Genetically distinct glioma stem-like cell xenografts established from paired glioblastoma samples harvested before and after molecularly targeted therapy

**DOI:** 10.1038/s41598-018-37437-2

**Published:** 2019-01-15

**Authors:** Shota Tanaka, Samantha Luk, Juri Kiyokawa, Maristela L. Onozato, A. John Iafrate, Khalid Shah, Robert L. Martuza, Samuel D. Rabkin, Tracy T. Batchelor, Daniel P. Cahill, Andrew S. Chi, Hiroaki Wakimoto

**Affiliations:** 1 Divisions of Neuro-Oncology and Hematology/Oncology, Harvard Medical School, Boston, Massachusetts USA; 2 Department of Neurosurgery, Harvard Medical School, Boston, Massachusetts USA; 3 Department of Pathology, Massachusetts General Hospital, Harvard Medical School, Boston, Massachusetts USA; 4 Department of Neurosurgery, The University of Tokyo Hospital, Tokyo, Japan; 5 Center for Stem Cell Therapeutics and Imaging, Department of Neurosurgery, Brigham and Women’s Hospital, Harvard medical School, Boston, Massachusetts USA; 6 Present Address: Laura and Isaac Perlmutter Cancer Center, NYU Langone Health and NYU School of Medicine, New York, NY USA

## Abstract

Intratumoural heterogeneity underlies tumour escape from molecularly targeted therapy in glioblastoma. A cell-based model preserving the evolving molecular profiles of a tumour during treatment is key to understanding the recurrence mechanisms and development of strategies to overcome resistance. In this study, we established a matched pair of glioblastoma stem-like cell (GSC) cultures from patient glioblastoma samples before and after epidermal growth factor receptor (EGFR)-targeted therapy. A patient with recurrent glioblastoma (MGG70R) harboring focal, high-level *EGFR* amplification received the irreversible EGFR tyrosine kinase inhibitor dacomitinib. The tumour that subsequently recurred (MGG70RR) showed diploid *EGFR*, suggesting inhibitor-mediated elimination of *EGFR*-amplified tumour cells and propagation of *EGFR* non-amplified cell subpopulations. The MGG70R-GSC line established from MGG70R formed xenografts retaining *EGFR* amplification and EGFR overexpression, while MGG70RR-GSC established from MGG70RR generated tumours that lacked *EGFR* amplification and EGFR overexpression. MGG70R-GSC-derived intracranial xenografts were more proliferative than MGG70RR-GSC xenografts, which had upregulated mesenchymal markers, mirroring the pathological observation in the corresponding patient tumours. *In vitro* MGG70R-GSC was more sensitive to EGFR inhibitors than MGG70RR-GSC. Thus, these molecularly distinct GSC lines recapitulated the subpopulation alteration that occurred during glioblastoma evasion of targeted therapy, and offer a valuable model facilitating therapeutic development for recurrent glioblastoma.

## Introduction

Despite the standard treatment with resection, radiotherapy, and the alkylating agent temozolomide^1^, glioblastoma harbors a poor prognosis and remains a fatal disease for the vast majority of cases. Numerous molecularly targeted agents have been investigated in both the preclinical and clinical settings, including first-generation epidermal growth factor receptor (EGFR)-targeted agents such as gefitinib (Iressa®, AstraZeneca, London, UK), erlotinib (Tarceva®, Roche, Basel, Switzerland), and lapatinib (Tykerb®, GlaxoSmithKline, Brentford, UK)^2^, based on the high prevalence of aberrant EGFR activation in glioblastoma^3,4^. More recently, second-generation EGFR-targeted agents with irreversible inhibition and better penetration into the brain have been developed including dacomitinib (PF-00299804) (Pfizer, New York, NY)^5,6^. Dacomitinib is active against glioblastoma in preclinical studies^7,8^ and has been tested in two clinical trials (NCT01112527, NCT01520870). Of note, patient accrual in these two trials was restricted to those with EGFR gene amplification in archival tumour specimens, with an expectation of their better response to PF-00299804^9^. The latter phase II trial (NCT01520870) reported a limited activity of the drug in recurrent glioblastoma with *EGFR* amplification, although a minor fraction of patients, 4 of 49 (8.2%), had durable ( > 6 months) response^10^.

Molecularly targeted agents have thus far been ineffective in the treatment of glioblastoma. Possible escape mechanisms include intratumoural heterogeneity^11–13^, loss of target gene expression and activation of redundant signaling pathways^14,15^. Elucidating these resistance mechanisms in more detail is critical for future studies of second-generation molecularly targeted agents. Our previous studies demonstrated that glioma stem-like cell (GSC)-enriched neurospheres phenotypically and genotypically recapitulate the patient tumours from which they were derived^16,17^. In this study, we established GSC neurospheres from patient tumour samples harvested before and after treatment with an EGFR-targeted agent, and analysed the molecular and biological characteristics that the GSC and patient tumour specimens exhibited pre- and post-treatment with this targeted drug.

## Results

### Phenotypic and genotypic comparison of paired patient tumour samples

**Figure 1:**
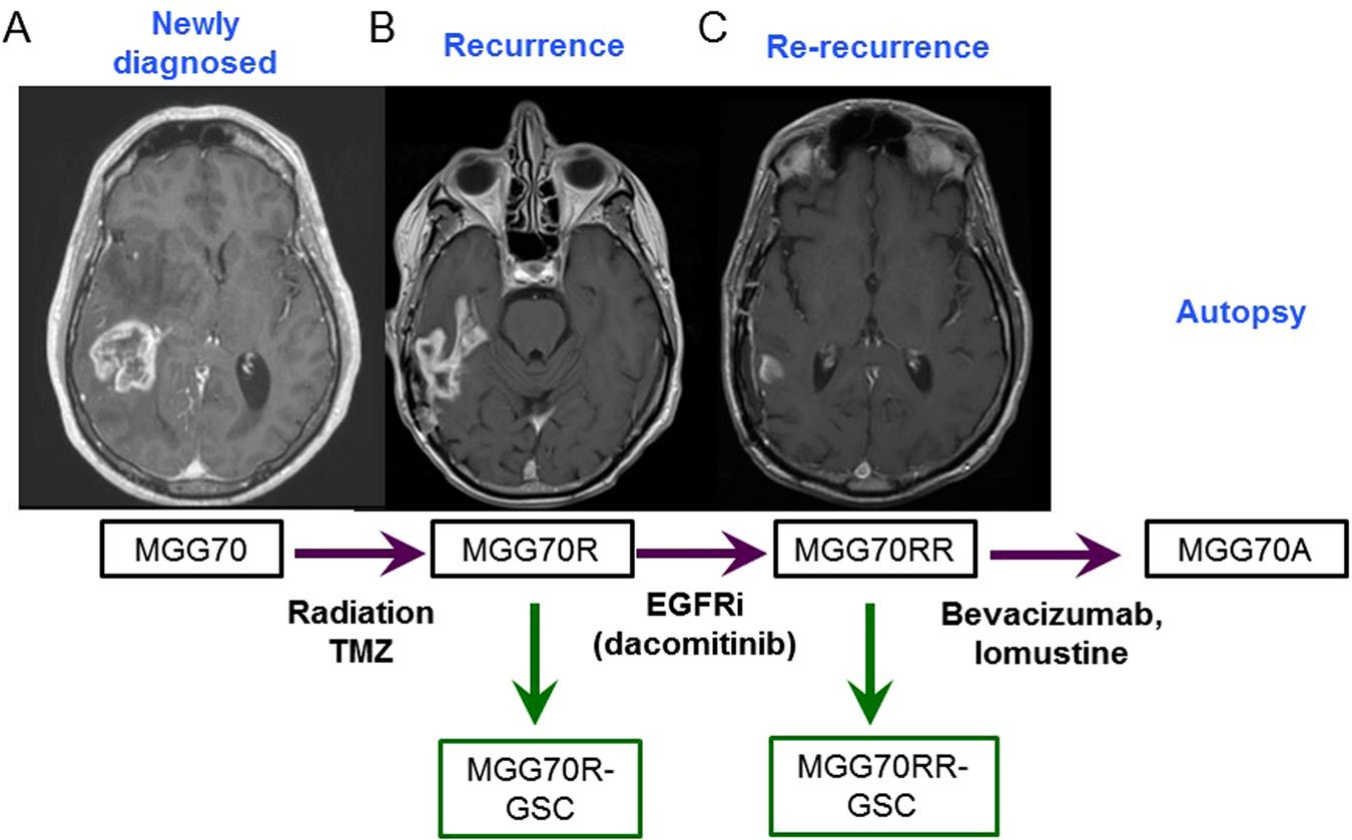
MRI and schema of clinical course of the patient GBM and glioma stem-like cell establishment. (**A**) The newly diagnosed tumour was located in the right posterior temporal lobe associated with perifocal edema. This was subtotally resected, followed by radiotherapy and chemotherapy with temozolomide (TMZ). (**B**) The tumour recurred after one year (MGG70R), which was resected. The patient was then treated with an EGFR inhibitor, dacomitinib. (**C**) After two months with two cycles of dacomitinib, the tumour recurred again (MGG70RR), which was surgically removed. After subsequent treatment with Bevacizumab and lomustine, the patient passed away and autopsy was performed. 70R-GSC and 70RR-GSC were established from the surgical specimens of MGG70R and MGG70RR, respectively.

**Figure 2:**
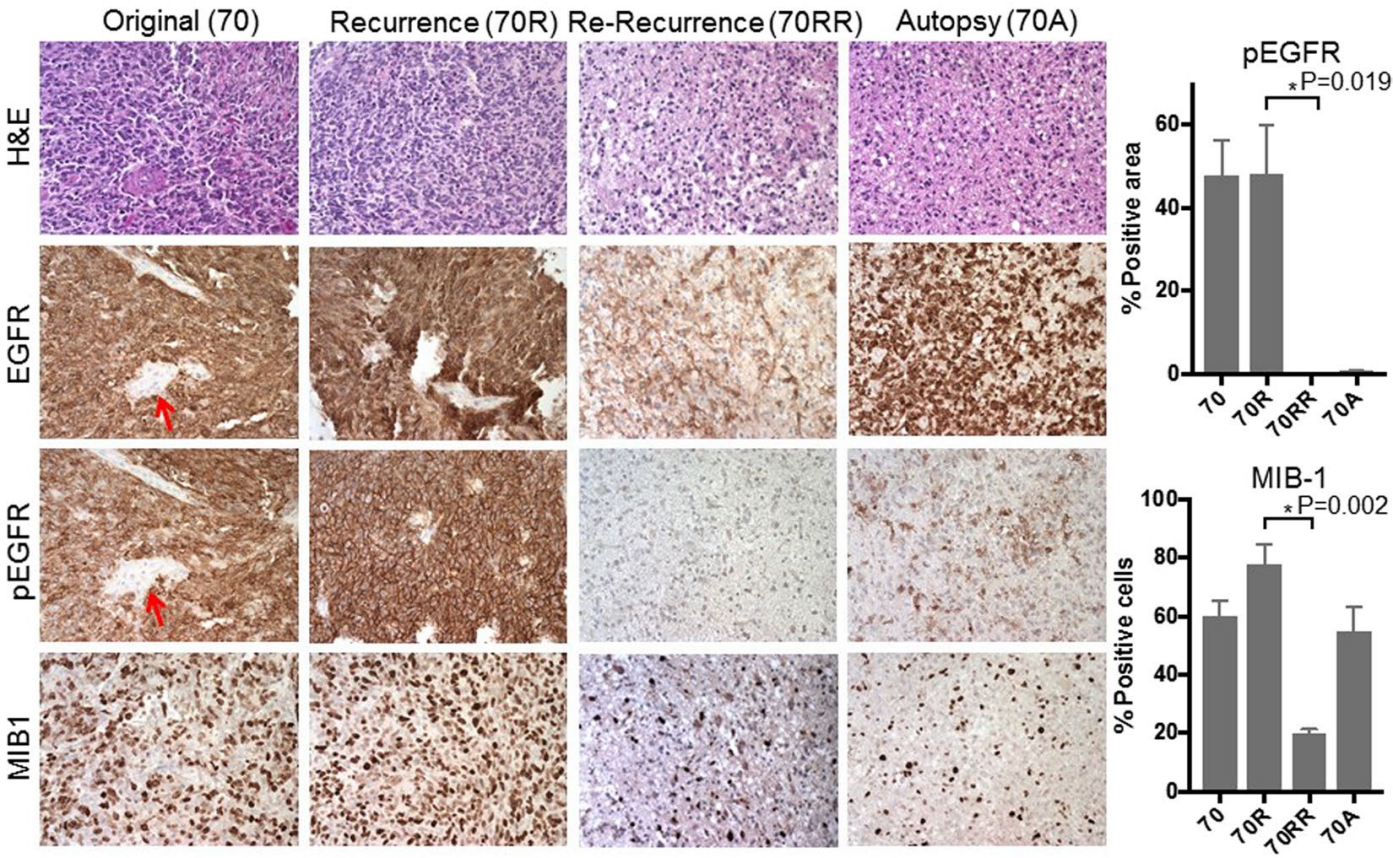
Histopathological analysis reveals drastic changes in EGFR and MIB1 status in re-recurrent GBM after EGFR inhibitor therapy. Microscopic pictures of H&E staining (top row) and IHC for EGFR (2^nd^ row), phospho-EGFR (Tyr1068, 3^rd^ row) and MIB-1 (Ki-67, the bottom row) for, from left to right column, original tumour MGG70 (70), recurrent tumour MGG70R (70R), re-recurrent tumour MGG70RR (70RR), and autopsy material MGG70A (70A). Original magnification, x200. Note negative staining of EGFR and phospho-EGFR in tumour-associated blood vessels (arrows). pEGFR positivity (% area) and MIB-1 labeling indices of each tumour are plotted on the right.

**Figure 3:**
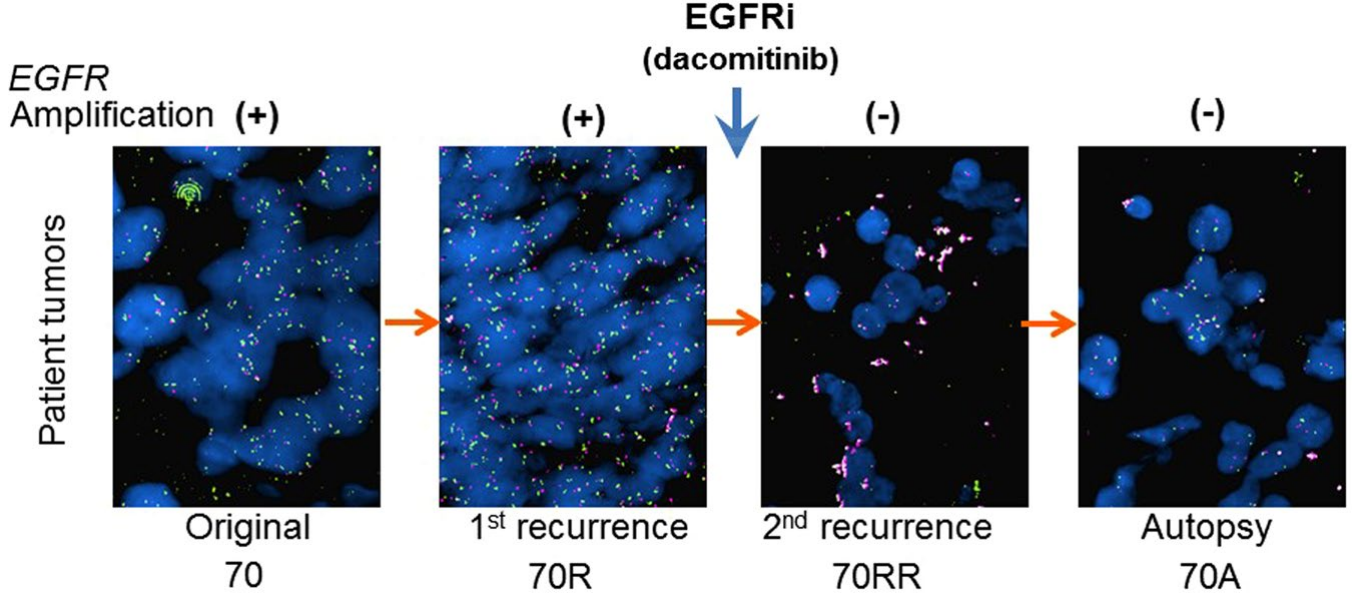
EGFR inhibition resulted in elimination of *EGFR*-amplified populations. FISH images of the patient tumours, with *EGFR* probe in green and centromere 7 (CEN7) control probe in red. From left to right, panel represents the original tumour MGG70 (70), the first recurrent tumour before dacomitinib treatment MGG70R (70R), the re-recurrent tumour after dacomitinib treatment MGG70RR (70RR) and the autopsy material MGG70A (70A). Clumped amplification of *EGFR* is noted in 70 and 70 R, but not in 70RR and 70 A.

Using FFPE samples of the original tumour, recurrent tumour, re-recurrent tumour and autopsy of this glioblastoma case (Fig. 1), we first characterized histopathological phenotypes of the tumours. Immunohistochemical analysis showed that MGG70R (pre-dacomitinib tumour) had diffuse and intense immunopositivity for EGFR and its activated form phospho-EGFR, a very similar staining pattern to that observed in the original tumour MGG70 (Fig. 2, Supplementary Fig. S1). In contrast to these tumours (MGG70 and MGG70R), the expression of EGFR and phospho-EGFR was substantially decreased in the post-dacomitinib tumour MGG70RR (Fig. 2). MIB-1 (Ki-67) staining revealed that MGG70RR exhibited a significantly lower proliferative rate compared to MGG70 and MGG70R (*P* = 0.03) (Fig. 2). Genetically, FISH analysis showed focal *EGFR* amplification in the newly diagnosed tumour MGG70, which was retained at a higher level in the recurrent MGG70R specimen (Fig. 3), suggesting that the treatment with radiotherapy and temozolomide did not preferentially target cell populations harboring amplified *EGFR*. In contrast, all tumour cells had diploid *EGFR* signals in the post-dacomitinib MGG70RR (Fig. 3). In the brain obtained at autopsy, there were scattered foci with relatively strong immunostaining of EGFR/phospho-EGFR (Fig. 2), but FISH analysis did not detect any cells with *EGFR* amplification (Fig. 3). Of note, gene amplification of other receptor tyrosine kinases (RTKs) such as platelet-derived growth factor receptor (*PDGFR*) and *MET* was not noted in any of the tumour samples (Supplementary Fig. S2). Thus, in this glioblastoma patient, prominent phenotypic and genotypic changes, most notably the elimination of *EGFR*-amplified tumour cells, occurred after targeted treatment with an EGFR tyrosine kinase inhibitor.

### Phenotypic and genotypic characterization of GSC-derived xenografts and comparison to patient tumour specimens

**Figure 4:**
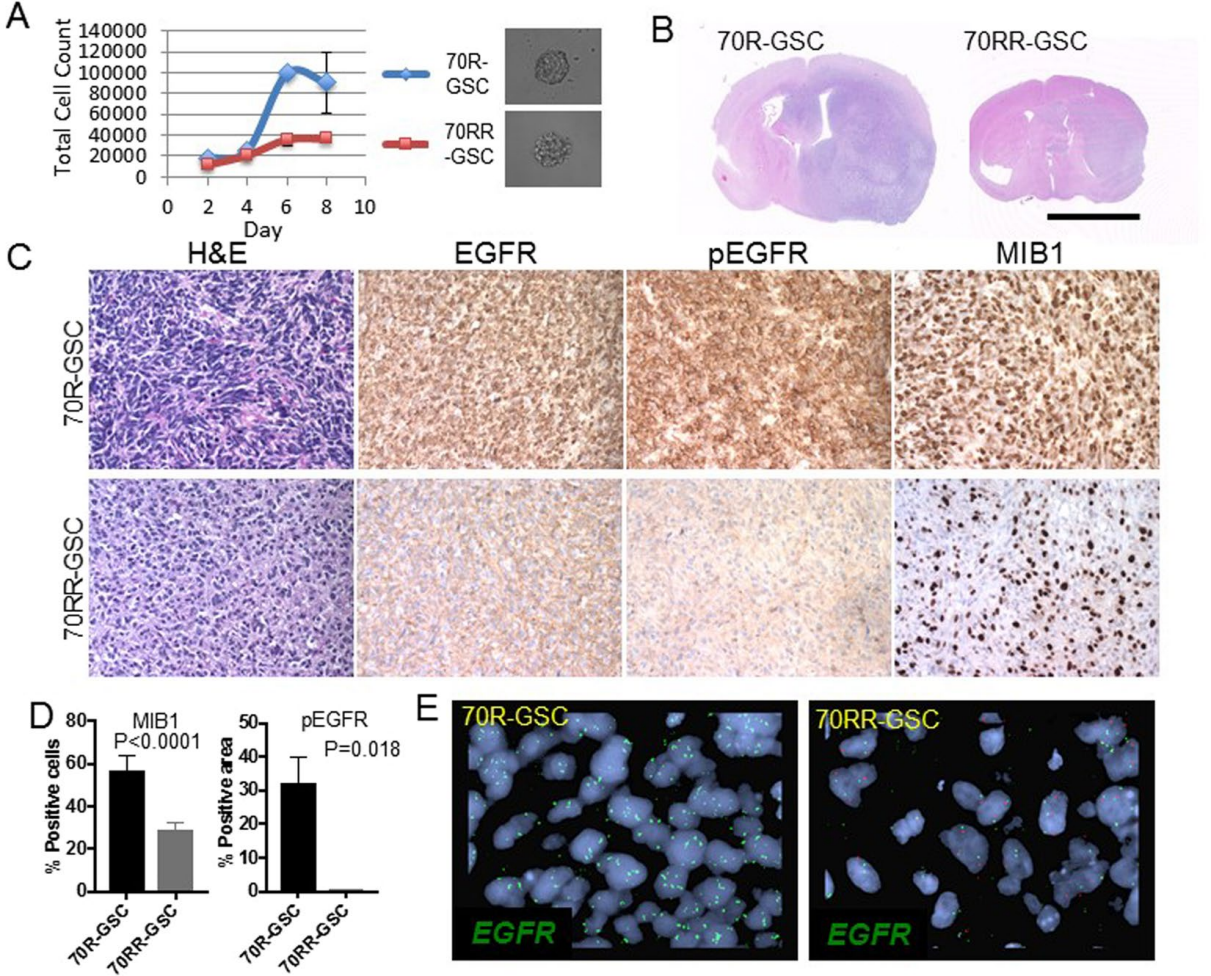
Paired GSC orthotopic xenografts recapitulate the molecular and phenotypic characteristics of patient tumours. (**A**) Cell counting assay showing different growth kinetics of MGG70R-GSCs (70R-GSC) and MGG70RR-GSCs (70RR-GSC) in culture. Shown right are representative microscopic pictures of a neurosphere generated from a single GSC. (**B**) H&E staining of coronal sections of the brains bearing GSC-derived orthotopic tumours. Scale bar, 5 mm. (**C**) H&E staining and IHC for EGFR, phospho-EGFR, and Ki-67 (MIB-1) in orthotopic GSC xenografts. Top row, MGG70R-GSC tumours. Bottom row, MGG70RR-GSC tumours. (**D**) MIB-1 labeling indices and pEGFR immunopositivity (% area) of MGG70R-GSC and MGG70RR-GSC tumours. (**E**) FISH images of the GSC xenografts, with *EGFR* probe in green and CEN7 control probe in red. Left, MGG70R (pre-dacomitinib) GSC tumour. Right, MGG70RR (post-dacomitinib) GSC tumour.

We successfully established neurosphere cultures from pre- and post-dacomitinib patient tumour samples (MGG70R and MGG70RR) (Figs 1, 4A). MGG70R-GSC and MGG70RR-GSC had comparable abilities to generate a sphere from a single cell. However, cell proliferation assays demonstrated that MGG70R-GSC proliferated at a faster rate than MGG70RR-GSC (Fig. 4A). Both MGG70R-GSC and MGG70RR-GSC were able to generate orthotopic xenografts in SCID mice (Fig. 4B). Although MGG70R-GSC as well as MGG70RR-GSC-derived intracranial tumours became lethal within a similar time frame (~2 months), the size of the brains harboring MGG70R-GSC xenografts were considerably larger than that of the brains with MGG70RR-GSC xenografts (Supplementary Fig. S3). H&E stain showed that the MGG70R-GSC tumour was much larger than MGG70RR-GSC tumour, causing a striking enlargement of the implanted hemisphere while both xenografts displayed a semi-invasive phenotype with signs of moderate invasiveness (Fig. 4B). In concordance with the aforementioned changes observed in the patient tumours, the post-dacomitinib MGG70RR-GSC xenografts exhibited reduced levels of EGFR/phospho-EGFR expression as well as reduced MIB-1 labeling index, i.e., the proliferation rate (*P* = 0.007), compared to the pre-dacomitinib MGG70R-GSC xenografts (Figs 4C,D). *EGFR* amplification was observed in the MGG70R-GSC-derived xenografts but not in the MGG70RR-GSC-derived xenografts, indicating that the GSC-generated xenografts preserved the *EGFR* status observed in the respective patient tumour specimens (Fig. 4E). These results show that GSC neurospheres established from pre- and post-dacomitinib treatment recapitulated the phenotypic and genotypic profiles of the corresponding tumour samples in the same patient.

### Phenotypic and molecular characterization of GSC neurospheres *in vitro*

**Figure 5:**
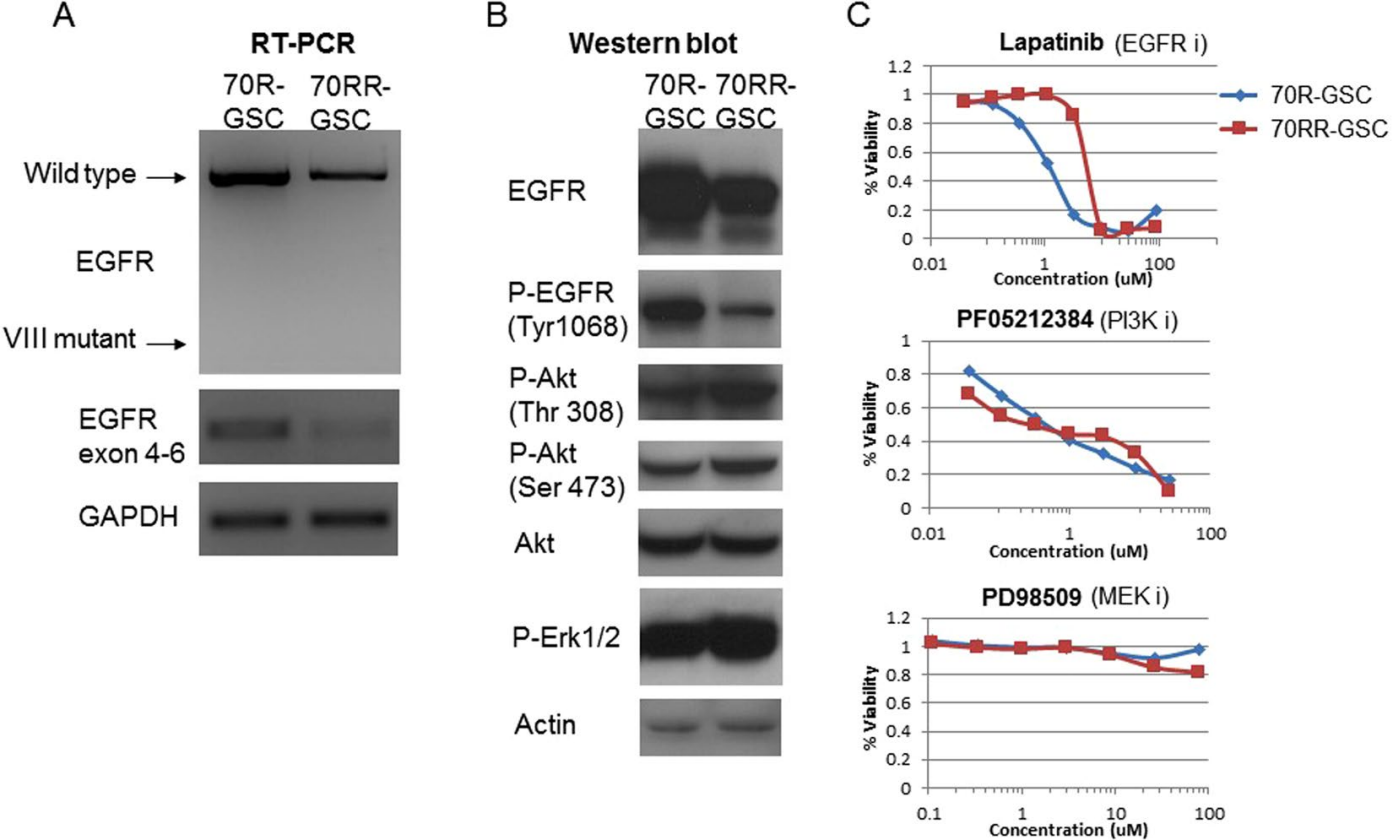
Pre-EGFR inhibitor GSCs overexpress EGFR and phospho-EGFR and are sensitive to EGFR inhibition. (**A**) RT-PCR showing *EGFR* expression in the paired GSCs, MGG70R-GSC (70R-GSC) and MGG70RR-GSC (70RR-GSC). GAPDH was used as control. (**B**) Western blotting showing EGFR, phospho-EGFR (Tyr1068), phospho-Akt (Thr308), phospho-Akt (Ser473), Akt, and phospho-Erk1/2 in 70R and 70RR GSCs. Actin served as control. (**C**) GSC sensitivities to molecular targeted agents. Cell viability assay was performed on the paired GSCs after 3-day exposure to the EGFR inhibitor lapatinib (upper), the PI3K inhibitor PF-05212384 (middle) and the MEK inhibitor PD98509 (bottom).

We further molecularly characterized patient-derived GSCs *in vitro*. Semi-quantitative RT-PCR and western blotting showed that MGG70R-GSC overexpressed wild-type EGFR at both the transcript and protein levels and had a higher amount of phospho-EGFR in comparison to MGG70RR-GSC (Fig. 5A,B). However, western analysis of the PI3K pathway downstream of EGFR revealed that the status of Akt phosphorylation (at Thr308 and Ser473) was comparable or slightly more pronounced in MGG70RR-GSC (Fig. 5B). Similarly, activation of Erk1/2, a MAPK pathway signal transducer, was present in the paired GSC lines, but was higher in MGG70RR-GSC (Fig. 5B).

### Sensitivities of the paired GSC neurospheres to different pathway inhibitors *in vitro*

We sought to determine the sensitivities of the paired GSC neurospheres to small molecule inhibitors that target different oncogenic pathways. Based on the results of western blotting and immunohistochemistry, we selected an EGFR inhibitor (lapatinib), a PI3K inhibitor (PF-05212384), and a MEK inhibitor (PD98509). *In vitro* cell viability assays revealed that, as expected, the paired GSC neurospheres showed differential sensitivities to lapatinib; MGG70RR-GSC was significantly more resistant compared to MGG70R-GSC (Fig. 5C). However, both GSC lines displayed a comparable sensitivity to PF-05212384 with an EC50 value below 1 μM (Fig. 5C). Both GSC lines were resistant to PD98509 (Fig. 5C).

### Evaluation of mesenchymal markers and Met expression in the paired tumour specimens

**Figure 6:**
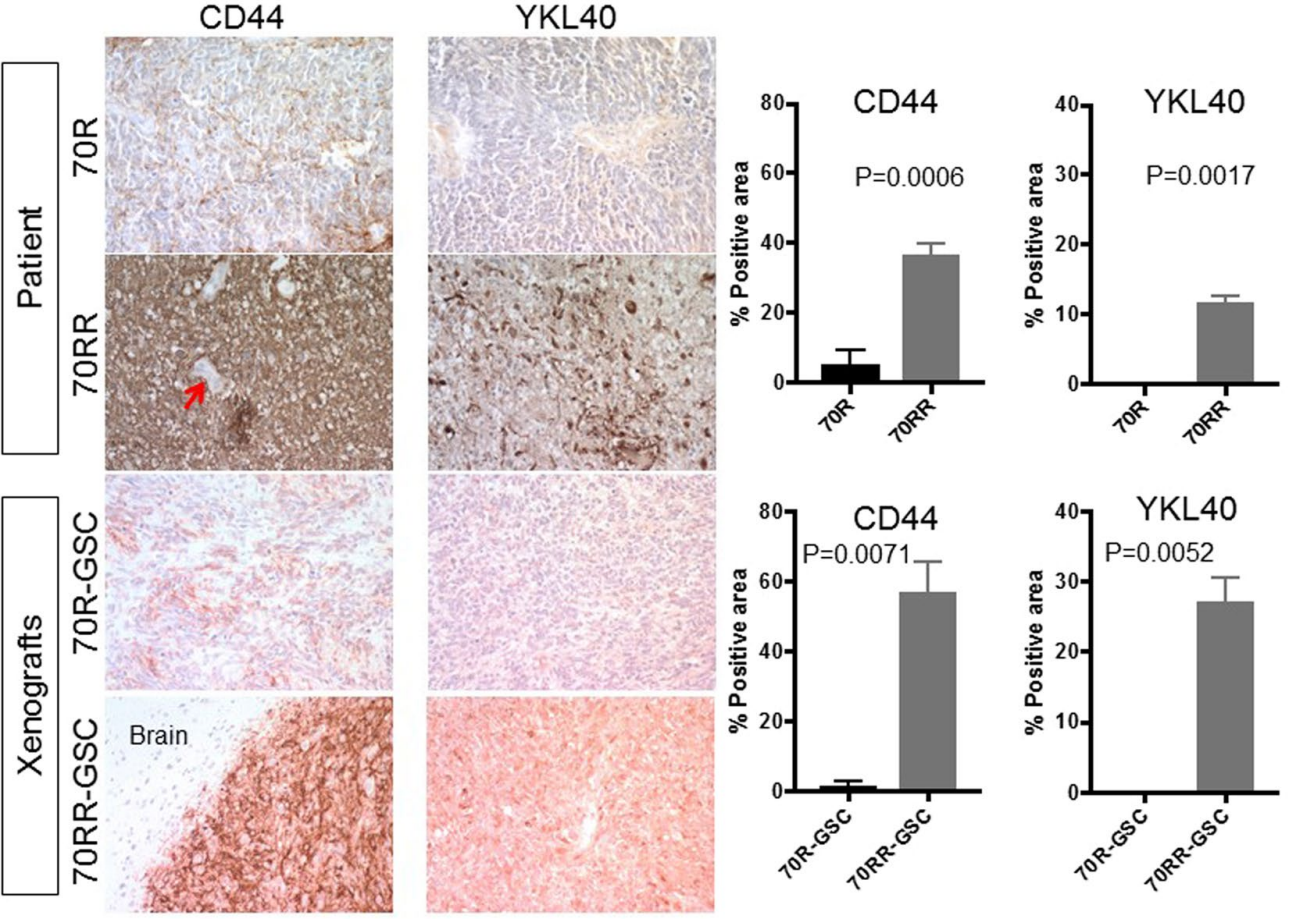
MGG70RR tumour and its GSC xenografts overexpress mesenchymal markers. Immunohistochemistry (IHC) for CD44 and YKL-40 in the patient tumours, MGG70R (70R) and MGG70RR (70RR), and the corresponding orthotopic GSC xenografts. Upper two rows, patient tumours. Lower two rows, xenografts. For CD44 IHC in MGG70RR-GSC tumours, a tumour-brain border area was depicted to demonstrate tumour-selective staining. Note negative staining of CD44 in tumour-associated blood vessels (arrow). Image quantification of IHC is shown on the right: upper plots, patient tumors; lower plots, xenografts.

Since epithelial to mesenchymal transition (EMT) is implicated in cancer progression and resistance^18,19^, we explored whether a similar process is involved in glioblastoma resistance to dacomitinib. We observed that the expression of the mesenchymal markers CD44 and YKL-40 was upregulated at the time of recurrence on dacomitinib (MGG70RR) and that this change was phenocopied in the GSC-derived xenografts (Fig. 6). Intratumoural microvascular density was also elevated in MGG70RR-GSC-derived intracranial tumours (Supplementary Fig. S4). Furthermore, since Met overexpression and *MET* amplification has been implicated in acquired resistance to EGFR inhibition^20,21^, we examined Met expression in MGG70R and MGG70RR patient tumour specimens and the respective GSC tumours. We found that Met was not expressed in any of these tumours (Supplementary Fig. S5A), suggesting that Met was not involved in the resistance of MGG70RR-GSC to EGFR inhibitors. Accordingly, both MGG70R-GSC and MGG70RR-GSC were resistant to the Met inhibitor crizotinib (Supplementary Fig. S5B).

## Discussion

Here we present a case of recurrent glioblastoma in which *EGFR* gene amplification present in the pre-treatment tumour subsequently disappeared in the tumour that progressed on targeted treatment with the EGFR tyrosine kinase inhibitor dacomitinib. We successfully established GSC neurospheres from the above longitudinal tumours and demonstrated that they recapitulated the molecular changes noted in the patient tumour specimens.

To the best of our knowledge, this is the first report in glioblastoma that documents loss of receptor tyrosine kinase gene amplification in patient’s tumour after direct molecularly targeted inhibition with a small molecule agent, an observation reported in other cancers^22,23^. EGFRvIII, a mutant form of EGFR, was eliminated on immunohistochemistry in the majority of glioblastomas after treatment with the anti-EGFRvIII peptide vaccine rindopepimut^24^. In contrast, we show that *EGFR* amplification present in the primary tumour was retained in the recurrent tumour after treatment with radiotherapy and temozolomide, in line with a recent report showing relative stability of *EGFR* amplification and EGFRvIII mutation within paired primary and recurrent glioblastomas^25,26^.

The pre-dacomitinib MGG70R contained focal clusters with *EGFR*-amplified cells, suggesting intratumoural heterogeneity, a hallmark of glioblastoma^11–13,27^. Loss of *EGFR* amplification in the post-dacomitinib tumour indicates differential efficacy of EGFR-targeting within a single tumour depending upon *EGFR* amplification status^28^; it appears that *EGFR*-amplified cells were selectively eliminated by irreversible EGFR inhibition with dacomitinib and the non-amplified cells that survived seeded the re-recurrent tumour. Darwinian selection of preexisting drug-resistant clones, or so-called “subpopulation switch” has been recognized in cancer^29^. The current case highlights intratumoural heterogeneity as a key mediator of tumour evasion^30,31^ and recurrence after molecularly targeted therapy.

The unique cell models based on the paired GSC-enriched neurosphere cultures that we established before and after targeted treatment allowed us to characterize their molecular signatures and *in vivo* phenotypes. FISH analyses on the paired GSC xenografts demonstrated amplified and non-amplified *EGFR* in pre- and post-dacomitinib tumours, respectively, which reflected the differential EGFR expression levels on immunohistochemistry. In addition, the cell proliferation marker, MIB-1 in the GSC xenografts corresponded to the patient tumours. Each of these GSC models thus mirrors the biological behavior of the patient tumour specimen from which the GSCs were derived, representing the respective stage of the tumour during the clinical course of tumour evolution. This is in line with previous work from our group and others demonstrating that the GSC-enriched neurosphere models faithfully recapitulate the molecular and phenotypic characteristics of original patient tumours^16,17,32–34^.

Since MGG70RR GSCs must have been derived from subpopulations that had survived dacomitinib, we expected that these GSCs would be resistant to EGFR inhibition. Indeed, the post-dacomitinib GSCs exhibited an elevated resistance to EGFR tyrosine kinase inhibitors compared to the pre-dacomitinib GSCs. This finding provides experimental support to our clinical observation that non-*EGFR*-amplified glioblastoma cells were preferentially resistant to dacomitinib. Interestingly, small molecule inhibitors of the major EGFR downstream signaling pathway, the PI3K-Akt axis, resulted in comparable viability reduction of the pre- and post-dacomitinib GSCs. Phosphorylation status of both Akt and Erk was comparable between the GSCs despite clearly reduced EGFR phosphorylation in post-dacomitinib GSCs. Thus, the loss of *EGFR* amplification rendered GSCs less dependent on the EGFR signaling for survival/proliferation, however did not alter the dependence on the hub pathway of PI3K-Akt. Compensatory activation of other ErbB family^35^ or alternative receptor tyrosine kinases such as Met^20,21,36^ through gain of gene amplification, and downstream signaling by acquired genetic alterations such as PTEN loss^37,38^ could contribute to the acquired resistance to EGFR tyrosine kinase inhibitors. Lack of *MET* amplification, Met and phosphorylated Met levels, and insensitivity to the Met inhibitor crizotinib in pre and post-dacomitinib GSCs support the notion that Met does not play a significant role in the emergent tumour resistance in the current case. Alternatively, EMT could contribute to the acquired resistance to EGFR inhibitors as implicated in lung cancer^18^. Indeed, we did observe increased expression of representative mesenchymal markers in the post-dacomitinib tumours, both in the patient and in the xenografts, as seen with other recurrent GSC xenografts^39^. Further study is necessary to determine whether an EMT-like phenomenon observed in relapsed glioblastoma is associated with resistance to targeted therapy.

Here we show that paired GSC neurospheres serve as a unique preclinical model that allows investigations of the genetic and biological evolution of tumours, and treatment evasion mechanisms in glioblastoma. However, whether the findings derived from the particular case are applicable beyond the single patient is currently uncertain, which represents the limitation of the current work. Future studies are necessary to clarify whether GSC cultures may provide a robust tool enabling selection of patients potentially receiving benefit from a certain molecularly targeted agent^40–42^. Resection of relapsed disease could bring opportunities for not only assessing pharmacodynamic treatment effect and molecular profiling, but also for isolation of GSCs to screen drugs potentially efficacious for recurrent disease.

## Conclusion

GSC neurospheres established from longitudinal patient glioblastoma samples before and after targeted treatment with the EGFR inhibitor dacomitinib recapitulated the molecular changes observed in the patient tumours. Paired GSCs before and after therapy serve as a powerful tool for understanding of tumour evolution through the clinical course of molecularly targeted therapy as well as for drug screening for the recurrent disease.

## Materials and Methods

All methods were performed in accordance with the relevant guidelines and regulations. The Institutional Review Board (IRB) at MGH approved the portion of this study involving a human participant. Clinical information and tumour specimens were collected under IRB-approved protocols and informed consent had been previously obtained from the patient. The animal procedures were approved by the Institutional Animal Care and Use Committee (IACUC) at MGH.

### Clinical course of an EGFR-amplified glioblastoma case and establishment of paired glioma stem-like cells

A 62-year-old, right-handed man presented with personality changes, fatigue, and a left hemiparesis and was found to have a heterogeneously enhancing mass in the right temporal lobe associated with surrounding edema (Fig. 1A). The tumour was subtotally resected and the pathological diagnosis was glioblastoma (patient tumour designated as MGG70). He received postoperative standard therapy with focal, fractionated radiotherapy combined with concurrent and adjuvant temozolomide. The tumour recurred after one year (Fig. 1B), which was resected (designated MGG70R). A sample of fresh surgical specimen was used to generate GSC culture, designated MGG70R-GSC. As the recurrent tumour harbored focal, high-level *EGFR* amplification, he was enrolled in a phase II trial of an oral, irreversible EGFR tyrosine kinase inhibitor, dacomitinib (NCT01112527). However, the glioblastoma progressed on MRI scans after two months of treatment with dacomitinib (Fig. 1C). At the time of this second recurrence the tumour was resected (designated MGG70RR). Another GSC culture, designated MGG70RR-GSC, was generated from this surgical specimen Despite further treatment with bevacizumab and lomustine, he expired 7 months later. An autopsy was obtained (tumour designated MGG70A).

Tumour samples, MGG70R and 70RR, were sterilely obtained at surgery and were used to establish GSC-enriched neurospheres as described previously^43^. After enzymatic dissociation, cells were grown in Neurobasal medium (Invitrogen) supplemented with L-glutamine (3 mM; Mediatech), B27 supplement (Invitrogen), N2 supplement (Invitrogen), heparin (5 mg/mL; Sigma), EGF (20 ng/mL; R and D systems), and FGF2 (20 ng/mL; Peprotec). Neurospheres were passaged using TrypLE (Gibco).

### Pathology and immunohistochemistry

Hematoxylin and eosin (H&E) staining and immunohistochemistry were performed on formalin-fixed, paraffin-embedded (FFPE) tissues from MGG70, MGG70R, MGG70RR, and MGG70A, as described^16,43^. Primary antibodies used were MIB-1 (Dako, 1:150), EGFR, phosphorylated EGFR (phospho-EGFR), CD44, Mesenchymal-epithelial transition (Met) (all Cell Signaling, 1:500), and YKL40 (Quidel, 1:300). For antigen retrieval, microwave treatment in citrate buffer (for MIB-1), EDTA (1 mM, pH = 8, for EGFR and phosphorylated EGFR) and saponin (0.1%, for CD44 and YKL-40) were used. MIB-1 labeling index was calculated by counting MIB-1 positive cells and total cells (more than 1000 cells) in 4 randomly chosen high power fields within the different tumour specimens. Immuno-positivity of P-EGFR, CD44 and YKL40 was quantified by using Image J (NIH) in an unbiased manner and expressed as % positive area with SD.

### Fluorescence *in situ* hybridization

Fluorescence *in situ* hybridization (FISH) for *EGFR*, *PDGFRA and MET* was performed using BAC probes CTD-2113A18 (7p *EGFR* locus), RP11-114O6 (7q *MET* locus), RP11-819D11 (4q *PDGFRA* locus) and centromere 7 copy number control as described^11,44^. Cells were counted in at least 3 different high-power fields, and gene amplification was defined when gene/control probe copy number ratio was 2.0 or greater.

### Western blotting

Cells were lysed in RIPA buffer and lysates were analyzed via western blot as previously described^16^. The primary antibodies used were against EGFR, phospho-EGFR, Akt, phosphorylated-Akt (Ser473 & Thr308), extracellular signal-regulated kinase (Erk) 1/2, and phosphorylated-Erk 1/2 (all Cell Signaling, 1:1,000), and Actin (Sigma, 1: 5,000).

### Reverse transcription – polymerase chain reaction (RT-PCR)

Total RNA was extracted from cells using Trizol (Invitrogen). First strand cDNA was synthesized from 2 μg of RNA using Superscript 3 (Invitrogen). PCR was performed using Hotstar Taq polymerase (Qiagen) and the DNA samples were run on a 1% agarose gel containing ethidium bromide for visualization. The PCR primer sequences were: GAPDH (forward, CAATGACCCCTTCATTGACC; reverse, GACAAGCTTCCCGTTCTCAG), EGFR (forward, ATGCGACCCTCCGGGACG; reverse, ATTCCGTTACACACTTTGCGGC), and EGFR from exon 4 to 6 (forward, CATGTCGATGGACTTCCAGA; reverse, GGGCACAGATGATTTTGGTC).

### Cell proliferation assay

Dissociated cells were seeded at 20,000 cells per well to 24-well plates. Neurospheres were collected and dissociated after 2, 4, 6, and 8 days of culture, and viable cells were counted using trypan blue. The experiment was performed in triplicate.

### Mouse intracranial orthotopic xenograft models

GSC neurospheres were dissociated and 50,000 cells in 3 µL were stereotactically injected into the right striatum of 6–8 week-old female SCID mice as described previously^16,43^. Mice were sacrificed and brains removed when animals became symptomatic.

### Cell viability assay

Dissociated cells were seeded at 5,000 cells per well to 96-well plates; serially diluted drugs were added and cells were cultured for 3 days. Cell viability was measured by MTS cell viability assay with CellTiter 96 Aqueous One Solution (Promega, Madison, WI) to determine effective concentration 50 (EC50) values. Experiments were performed in triplicate. Targeted agents tested include the EGFR and HER2 inhibitor lapatinib (Tykerb®, GlaxoSmithKline, Brentford, UK), the phosphatidylinositol-3 kinase (PI3K) and mammalian target of rapamycin inhibitor PF-05212384 (gedatolisib) (Pfizer), the mitogen-activated protein kinase (MAPK)/Erk kinase (MEK) inhibitor PD98509 (Cell Signaling), and the mesenchymal-epithelial transition (Met) and anaplastic lymphoma kinase inhibitor crizotinib (Xalkori®, Pfizer, New York, NY).

### Statistical analyses

All statistical analyses were performed using JMP9.0 (SAS, Cary, NC). Two-tailed t-tests (unpaired) with Welch’s correction were employed to compare continuous variables between two groups. *P* values less than 0.05 were considered significant.

## Supplementary information

Supplementary Figures
