# Supplementary material for: Genetically distinct glioma stem-like cell xenografts established from paired glioblastoma samples harvested before and after molecularly targeted therapy
Source: Sci Rep. 2019 Jan 15;9:139. doi: 10.1038/s41598-018-37437-2 (PMC6333836; doi:10.1038/s41598-018-37437-2)

Genetically distinct glioma stem-like cell xenografts established from paired glioblastoma samples harvested before and after molecularly targeted therapy

Shota Tanaka[1,4*], Samantha Luk[2], Juri Kiyokawa[2], Maristela L. Onozato[3], A. John Iafrate[3], Khalid Shah[5], Robert L. Martuza[2], Samuel D. Rabkin[2], Tracy T. Batchelor[1], Daniel P. Cahill[2], Andrew S. Chi[1,6], and Hiroaki Wakimoto[2*]

[1]Divisions of Neuro-Oncology and Hematology/Oncology, [2]Department of Neurosurgery, and [3]Department of Pathology, Massachusetts General Hospital, Harvard Medical School, Boston, Massachusetts USA

[4]Department of Neurosurgery, The University of Tokyo Hospital, Tokyo Japan

[5]Center for Stem Cell Therapeutics and Imaging, Department of Neurosurgery, Brigham and Women's Hospital, Harvard medical School, Boston, Massachusetts USA

[6]Current address: Laura and Isaac Perlmutter Cancer Center, NYU Langone Health and NYU School of Medicine, New York, NY YSA

*corresponding authors

Supplementary Figure S1. Tumour specific immunopositivity of EGFR and phospho-EGFR. H&E staining and Immunohistochemistry (IHC) for EGFR and phospho-EGFR in the newly diagnosed patient tumour MGG70. Arrows show neoplastic cell-dense areas intensely stained for EGFR and phospho-EGFR. Two panels below represent negative EGFR and phospho-EGFR IHC on the normal human brain indicative of the specificity of the antibodies.

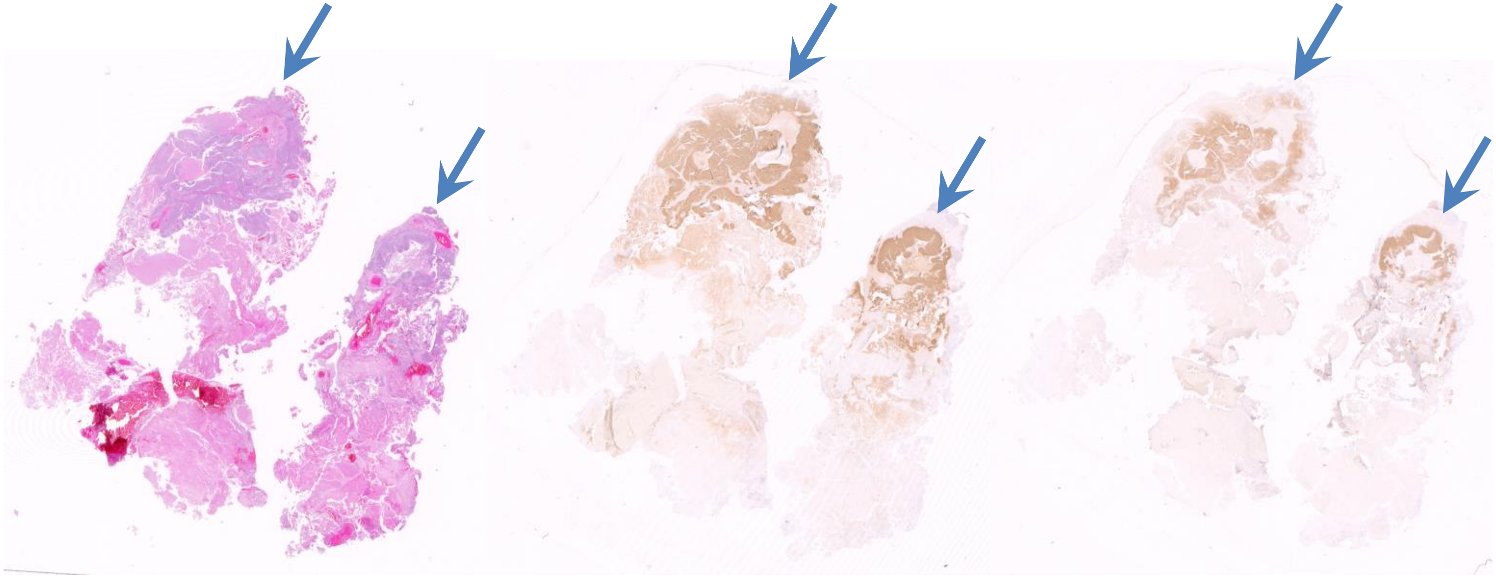

H and E EGFR P-EGFR (Y1068)

Normal human brain

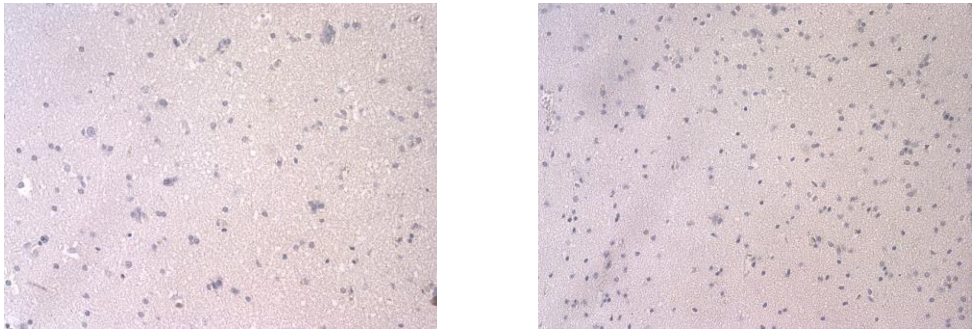

EGFR P-EGFR (Y1068)

Supplementary Figure S2. No amplification in *PDGFRA* and *MET*.
FISH showing polysomy and no amplification of *PDGFRA* and *MET* in both pre-dacomitinib MGG70R (70R) and post-dacomitinib MGG70RR (70RR) tumors in the patient. Bars, 5 mm.

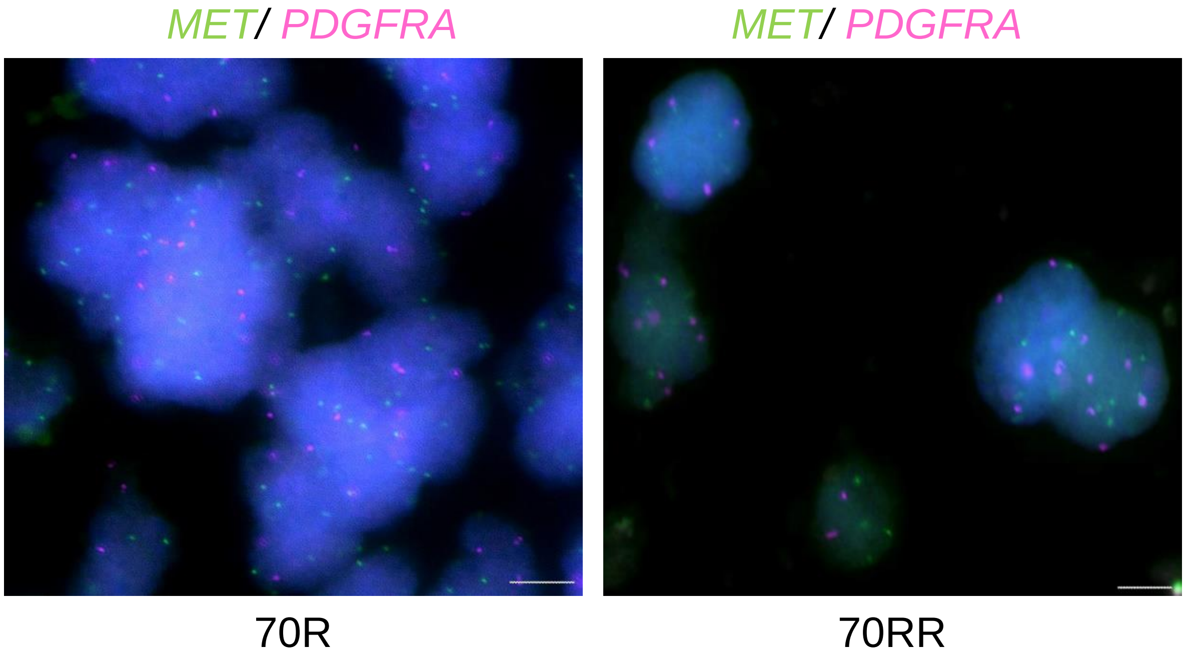

Supplementary Figure S3. Macroscopic appearance of the mouse brains bearing GSC xenografts.

Pictures of representative mouse brains (left, whole brain; right coronal cut through tumour region) bearing MGG70R-GSC tumour (left two panels, 70R-GSC) and MGG70RR-GSC tumour (right two panels, 70RR-GSC). Scale bar, 5mm.

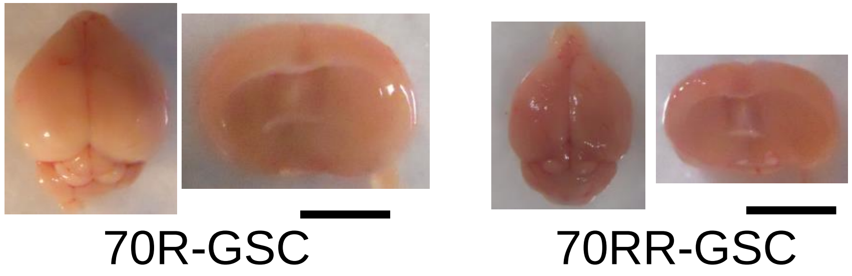

Supplementary Figure S4. Increased microvascular density in re-recurrent GSC orthotopic xenografts.

Immunohistochemistry for endothelial marker CD34 in orthotopic MGG70R-GSC (70R-GSC) and MGG70RR-GSC xenografts (70RR-GSC). Arrows depicting positive staining of micro-vasculature. Shown below is a quantitation of CD34-positive blood vessel densities comparing MGG70R and MGG70RR GSC tumours.

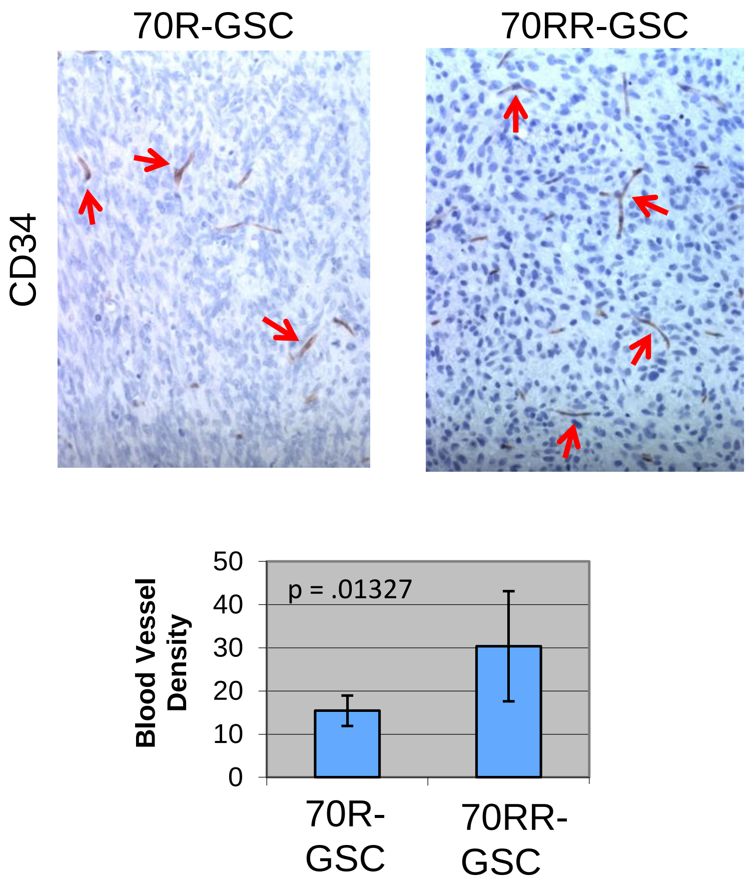

Supplementary Figure S5. Met is immunonegative in MGG70R and 70RR patient tumours and GSC xenografts.

(**A**) Met immunohistochemistry in patient tumours (top row) and the respective orthotopic GSC xenografts (bottom row). A glioblastoma known to have *MET* amplification, and its GSC tumour, served as positive controls. (**B**) Sensitivities of MGG70R-GSC (70R-GSC) and MGG70RR-GSC (70RR-GSC) to the Met inhibitor crizotinib. Cell viability assay was performed as in Fig. 5C.

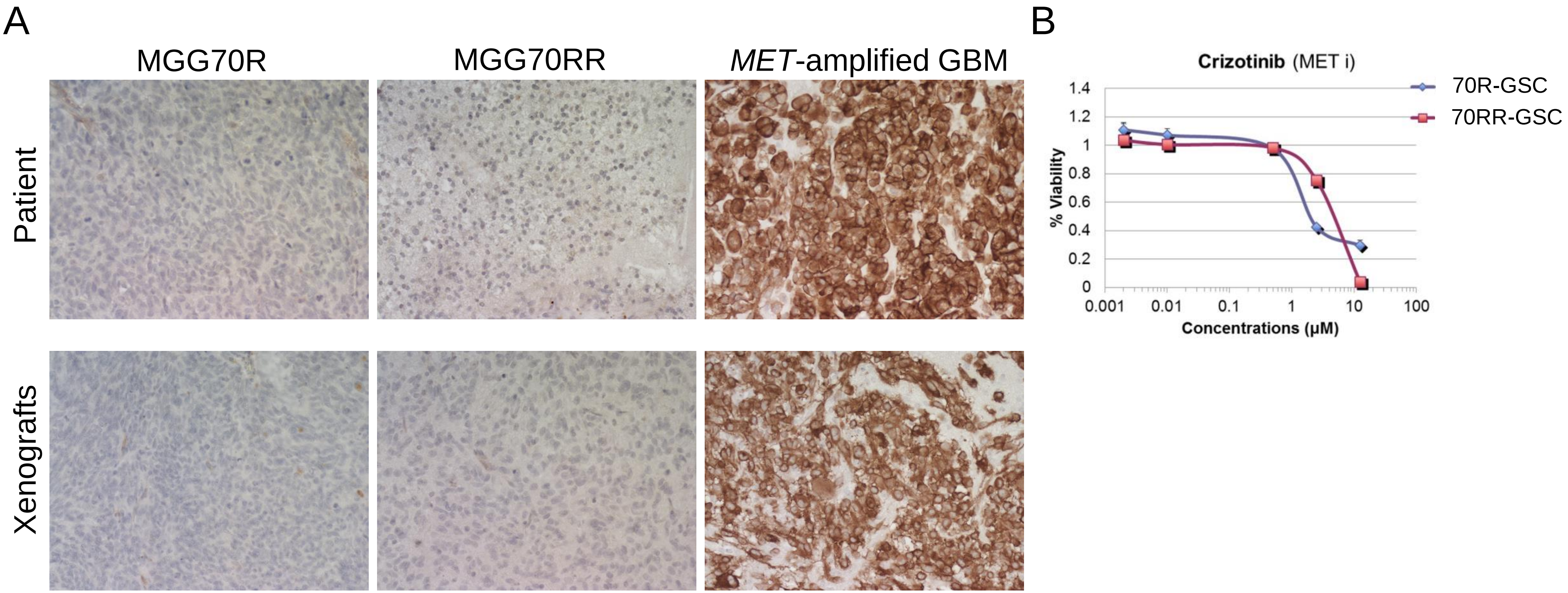

Supplement: Supplementary file 1 — Supplementary Figures [file 41598_2018_37437_MOESM1_ESM.pdf]
